# Accuracy of a Web-Based Time-Use Diary (MEDAL) in Assessing Children’s Meal Intakes With Food Photography by Parents as Reference: Instrument Validation Study

**DOI:** 10.2196/53461

**Published:** 2024-05-07

**Authors:** Kar Mun Chong, Airu Chia, Nur Syahirah Shah Budin, Bee Koon Poh, Nor Aini Jamil, Denise Koh, Mary Foong-Fong Chong, Jyh Eiin Wong

**Affiliations:** 1 Center for Community Health Studies (ReaCH) Faculty of Health Sciences Universiti Kebangsaan Malaysia Kuala Lumpur Malaysia; 2 Saw Swee Hock School of Public Health National University of Singapore Singapore Singapore; 3 Centre of Community Education & Wellbeing Faculty of Education Universiti Kebangsaan Malaysia Selangor Malaysia

**Keywords:** children, dietary intake, time-use diary, food photography, accuracy, mobile phone

## Abstract

**Background:**

My E-Diary for Activities and Lifestyle (MEDAL) is a web-based time-use diary developed to assess the diet and movement behaviors of Asian school children.

**Objective:**

This study aims to determine the accuracy of MEDAL in assessing the dietary intake of Malaysian school children, using photographs of the children’s meals taken by their parents as an objective reference.

**Methods:**

A convenience sample of 46 children aged 10 to 11 years recorded their daily meals in MEDAL for 4 days (2 weekdays and 2 weekend days). Their parents took photographs of the meals and snacks of their children before and after consumption during the 4-day period and sent them along with a brief description of food and drinks consumed via an instant SMS text messaging app. The accuracy of the children’s reports of the food they had consumed was determined by comparing their MEDAL reports to the photographs of the food sent by their parents.

**Results:**

Overall, the match, omission, and intrusion rates were 62% (IQR 46%-86%), 39% (IQR 16%-55%), and 20% (IQR 6%-44%), respectively. Carbohydrate-based items from the food categories “rice and porridge”; “breads, spreads, and cereals”; and “noodles, pasta, and potatoes” were reported most accurately (total match rates: 68%-76%). “Snack and dessert” items were omitted most often (omission rate: 54%). Furthermore, side dishes from “vegetables and mushrooms,” “eggs and tofu,” “meat and fish,” and “curry” food groups were often omitted (omission rates: 42%-46%). Items from “milk, cheese, and yogurt”; “snacks and desserts”; and “drinks” food groups intruded most often (intrusion rates: 37%-46%). Compared to the items reported by the boys, those reported by the girls had higher match rates (69% vs 53%) and lesser omission rates (31% vs 49%; *P*=.03, respectively).

**Conclusions:**

In conclusion, children aged 10 to 11 years can self-report all their meals in MEDAL, although some items are omitted or intruded. Therefore, MEDAL is a tool that can be used to assess the dietary intake of Malaysian school children.

## Introduction

### Background

Healthy dietary patterns developed during childhood and adolescence may track into adulthood [1]. Understanding children’s dietary patterns is crucial when guiding them to develop healthy eating habits from an early age. However, measuring the diets of school children has always been challenging, as doing so relies on the children’s cognitive abilities and abilities to recall dietary intake as well as estimate and indicate portion size [2,3]. While parents can be the primary proxy reporter, they may not be aware of what their child consumes away from home [4]. When children are aged approximately 7 to 8 years, their ability to recall without assistance slowly develops but only for food consumed in the past 24 hours [5], while their ability to remember and estimate portion size is still limited [6]. A study conducted in Malaysia showed that children aged 7 to 9 years can self-report their dietary intake for lunch accurately, without proxy assistance [7]. At the age of 8 to 12 years, children can self-report food intake as reliably as their parents [8]. However, self-reporting of food intake is tedious and time-consuming, which sometimes hamper the retrieval of dietary information from children [9]. Assessment tools that are intuitive, quick and simple to use, flexible, fun, engaging, nonintrusive, and age-appropriate can elicit the cooperation of children [9]. Therefore, to encourage and motivate children to self-report dietary assessment protocol, a novel assessment method may enhance the recruitment and completion rates while maintaining an acceptable level of data accuracy [2,10].

Children may find technology-based dietary assessment methods more attractive and appealing than paper-based methods [11]. The web-based dietary assessment method has proved to be a practical way of assessing children’s dietary intake [12,13]. In Singapore, a web-based time-use diary, My E-Diary for Activities and Lifestyle (MEDAL), has been developed to assess the diet and movement behaviors of school children aged ≥10 years [14]. MEDAL includes food options that are popular with the major ethnic groups in Asia and also captures information on food portions, location of activities, and concurrent activities performed by the children [14]. As Malaysia shares a border with Singapore and the Malaysian population exhibits similar food preferences and lifestyle behaviors, it is thought that MEDAL can be used by them. Therefore, MEDAL has been customized for Malaysian school children, with the addition of typical foods and beverages, as well as various activities practiced by Malaysian school children (eg, Solat [Muslim prayer], standing prayer, sitting or kneeling prayer, tuition classes and music lessons, track-and-field activities, and exercise).

The reference method in validating self-reported dietary intake is direct observation, where the agreement rates (match, omission, and intrusion rates) among direct observation, web-based food questionnaires [15,16], and web-based food records [17] are compared. This method provides precise information on the types and amount of food intake, with acceptable accuracy and good reliability [18]. However, these validation studies, which use direct observation as a reference method, only validate a single meal, while validation studies should include all meals, as the food items consumed vary with meal types, locations of consumption, and options available. The previous study conducted in Singapore [14] demonstrated that children could use MEDAL to self-report food intake for 1 meal during school recess but did not determine the accuracy of MEDAL to self-report food intake for all meals.

In addition, direct observation was usually conducted by trained researchers who had undergone extensive training in the visual estimation of food consumption [18]. Therefore, direct observation can be labor-intensive and subject to reporting errors by the observer. In addition, direct observation may not be feasible during situations such as the COVID-19 pandemic when movement restriction or physical distancing hinders researchers from directly observing the children’s food intake. However, while children were restricted from attending school, their parents were able to observe their children’s food intake. To minimize the parents’ burden as proxy reporters through direct observation, food photography by parents was adopted in this study as an objective measure to determine the accuracy of MEDAL in assessing the dietary intake of school children.

### Objective

With this background, this study aimed to determine the accuracy of MEDAL in assessing the dietary intake of Malaysian school children, using photographs of their children’s meals taken by parents as objective references.

## Methods

### Ethical Considerations

The protocol of this study was approved by the Universiti Kebangsaan Malaysia Research Ethics Committee (JEP-2019-307). Permission to conduct the study was obtained from the Ministry of Education, Malaysia; the relevant state education departments; and the school principals. The school children participating in the study were provided with informed consent forms to obtain permission from their parents before the commencement of the study.

### Study Participants

This study was conducted in April 2021 (study 1) and October to December 2021 (study 2) during the COVID-19 pandemic. A total of 53 children were recruited using convenience sampling, with 25 (47%) children from 2 primary schools for study 1 and 28 (53%) children from 3 primary schools for study 2. The study 1 students attended school in person, while the study 2 students attended classes online at home due to the implementation of movement control orders.

Primary school children aged 10 to 11 years were recruited for this study. Students were eligible if they understood Malay, English, or Chinese and had access to a computer, laptop, or tablet with internet connectivity. In addition, their parents had to own a smartphone with an internet connection to take food photos before and after meals and send them to us via an instant SMS text messaging app (eg, WhatsApp or Telegram). Information on the participants’ accessibility to electronic devices with internet access at home and their parents’ accessibility to a smartphone with internet access were obtained in the consent form.

### Study Procedures

#### MEDAL Web-Based App

MEDAL (National University of Singapore) is a self-administered, web-based time-use diary that captures the diet and activities of school children aged 10 to 12 years in the Asian context [14]. MEDAL was customized for use among Malaysian school children by translating the text into the Malay and Chinese languages and adding food and drinks that were consumed and activities that were performed typically by Malaysian school children. Children were requested to record daily activities from the time they woke up until bedtime for 4 days. They could choose from 6 broad categories of activities: “wash up/brush teeth,” “eat and drink,” “traveling,” “nap/sleep,” “sitting/praying activities,” and “active activities.”

When children recorded an “eat and drink” activity, they could choose the food and drink items they had consumed from 14 main food and drink groups (eg, breads, spreads, and cereals; curry or curry with coconut gravy; drinks; eggs and tofu; fast food; fruits; meat and fish; milk, cheese, and yogurt; vegetables and mushrooms; noodles, pasta, and potatoes; rice and porridge; snacks and desserts; soups; and supplements). For each food and drink item selected, the children also chose the portion size from 4 pictorial options (presented simultaneously) that best matched the amount consumed [14]. The children could use the “others” textbox if the items consumed were not listed in MEDAL and reported the amount consumed from the following 4 options: 1/2 portion, 1 portion, 1.5 portions, or 2 portions [14].

#### Demonstration of the Use of MEDAL

In study 1, we conducted a demonstration session in the computer laboratories of the participants’ schools. We demonstrated how to log in and record diet and daily activities in MEDAL. After the demonstration, the participants recorded what they consumed and did for 2 weekdays and 2 weekend days. For the second day, the participants were allowed to use school computers during recess to record their diet and activities if they had not completed the first day’s recording, after which they would continue recording at home for the remaining days.

In study 2, students could not attend school physically because of a government-imposed movement control order. Thus, face-to-face demonstration sessions for the students were not possible, and web-based demonstration sessions via Google Meet (Google LLC) were conducted instead. We demonstrated how to log in and record diet and activity entries with a prerecorded video. After the demonstration, the participants continued recording their entries at home using their own tablets, laptops, or computers.

#### Food Photography by Parents

Before the commencement of the study, all parents from both study periods were provided with standard guidelines on how to photograph the food their children were going to consume. To ensure compliance, reminders to take food photographs were sent to the parents’ SMS text messaging app twice a day before breakfast and dinner. During the 4 days of the study period, parents were required to photograph all the food and drinks consumed by their children, before and after consumption, for all meals, that is, breakfast, lunch, dinner, and snacks.

In study 1, the parents photographed all meals consumed by the children during nonschool times, while we photographed the school recess meal for the non-Muslim participants. Most Muslim participants (16 out of 25, 64% children) could not eat and drink during school recess, as study 1 was conducted during the Ramadhan fasting period.

**Figure 1:**
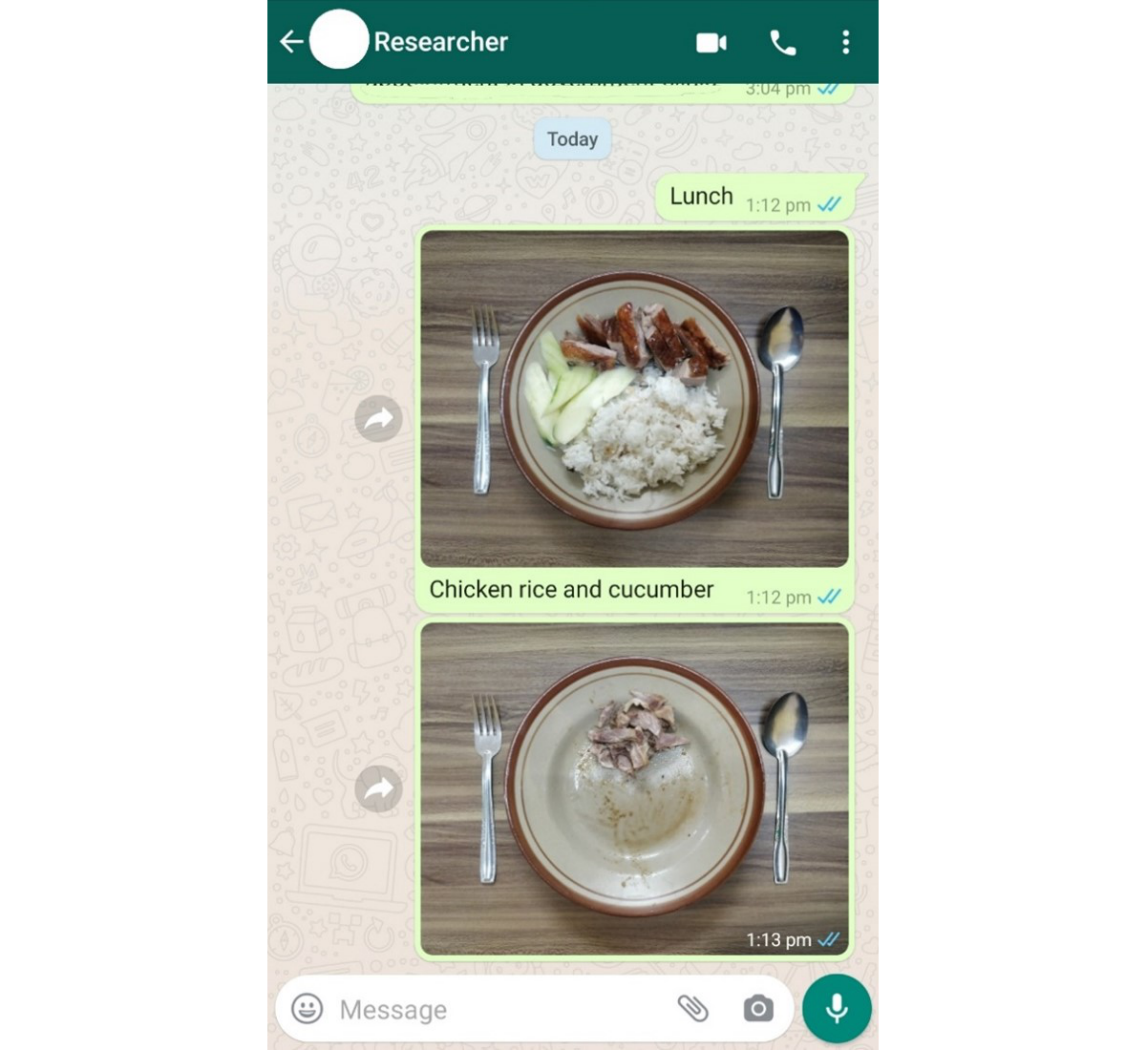
Photographs of the food taken before and after consumption, with a text description of the food consumed.

**Figure 2:**
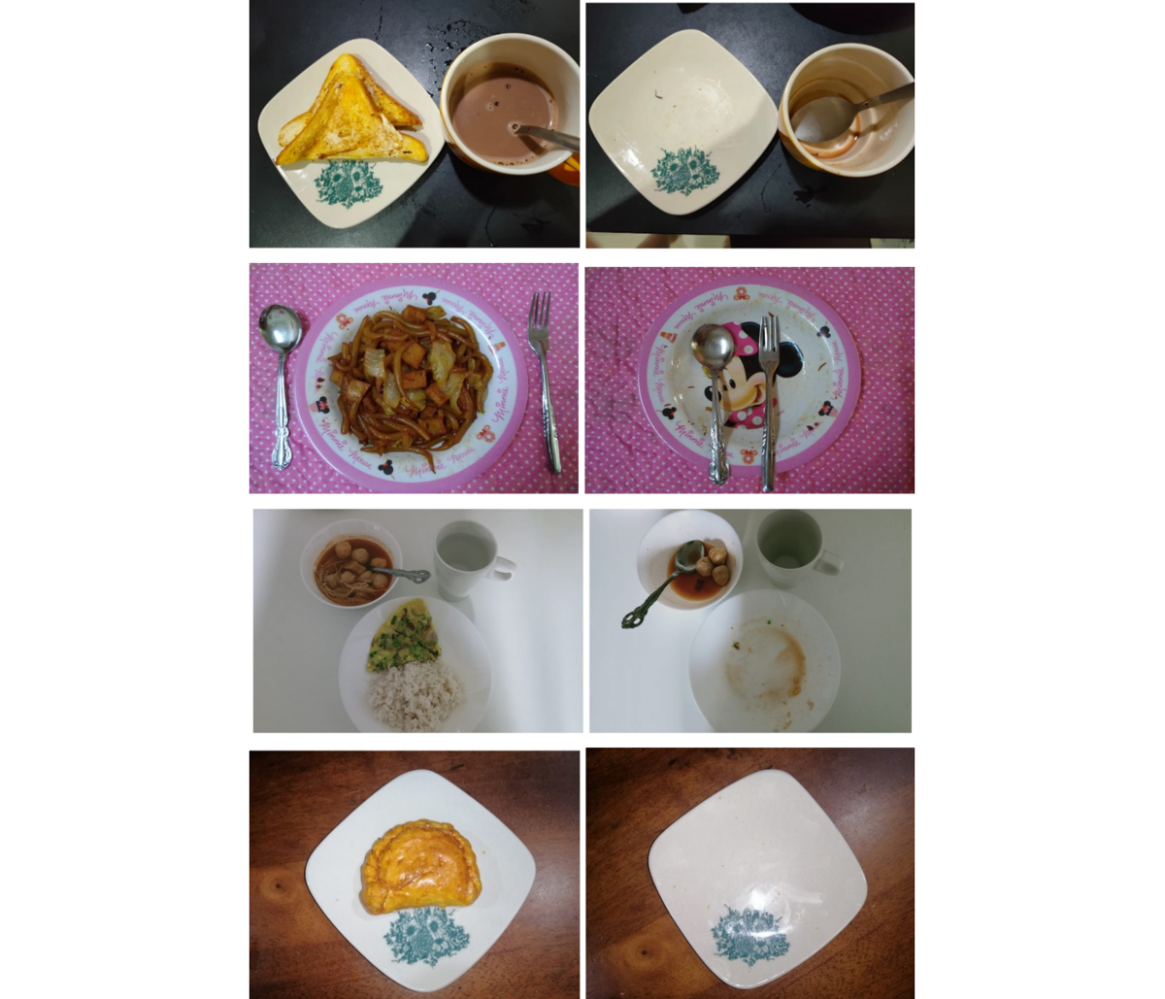
Examples of food photographs taken by parents before and after consumption of breakfast, lunch, dinner, and snacks.

In study 2, the parents photographed all meals consumed by the children as the children attended classes at home. Every day, parents sent photographs of the food and drinks before and after consumption, along with a brief description of what had been consumed, to us via an instant SMS text messaging app such as WhatsApp (Meta Platforms) or Telegram (Telegram FZ LLC). Figure 1 presents photographs with a description of the food and drink consumed. Figure 2 presents examples of food photographs for breakfast, lunch, dinner, and snacks taken before and after consumption.

#### Anthropometric Measurements

In study 1, we recorded children’s anthropometric measurements at schools. Height was measured using a portable Seca stadiometer (Seca 213, SECA, Hamburg, Germany) and weight using a Seca weighing scale (Seca 803, SECA, Hamburg, Germany). In study 2, anthropometric measurements of children were self-reported by parents due to the movement control order. Parents were asked to submit their children’s height and weight. Guidelines on how to make proper measurements were provided to the parents. School records of the participants’ most recent height and weight were used if home measurements were impossible.

BMI was calculated from body mass (kg) divided by the square of height (m^2^). BMI for age was calculated based on the World Health Organization growth reference for individuals aged 5 to 19 years [19]. *Z* scores for BMI for age were determined using the World Health Organization AnthroPlus (version 1.0.3) [20] software. The cutoff value for BMI for age for underweight was <−2 to −3 SD, overweight was >1 SD, and obesity was >2 SD. Values within > −2 SD and <1 SD were classified as normal weight.

### Data Processing

The accuracy of the child-reported food items was determined by comparing the self-reports of meal intake in MEDAL with the objective measures of food photographs taken by parents. Parents took photographs of the food before and after consumption for each meal taken by their children in the 4 days corresponding to the days of record in MEDAL. The meals included breakfast, lunch, dinner, and snacks or desserts. The food photographs taken by the parents for each meal were matched with the self-report by the children in MEDAL. The food photographs that matched the MEDAL diet entries for a meal were used for analysis. Missing food photographs or MEDAL entries were excluded from the analysis.

Food items in MEDAL include single items (eg, drinks, snacks, or desserts); composite meals consisting of staple food items with ingredients from the various food groups (eg, fried noodles); and components of mixed meals consisting of staple food items (eg, white rice) with side dishes (eg, steamed meat) [21]. Food items were classified into 14 food groups.

The accuracy of self-reported meals was determined by comparing the self-reported food items in MEDAL to the food items depicted as consumed in the photographs sent by the parents before and after consumption and classifying them as matches, omissions, and intrusion rates [17].

An “exact match” refers to a situation in which the food item self-reported by a child in MEDAL matches, to the maximum extent, the food item depicted in the reference photo provided by the parent. An “approximate match” refers to a situation in which the food item self-reported by a child in MEDAL matches with slight variations to the food item depicted in the reference photo. For example, a parent provides a reference photo of a glass of full cream milk their child had consumed. However, the child self-reported having consumed low-fat milk in MEDAL. The 2 food items are not identical but are recognized as similar, as they belong to the milk category. An “intrusion” refers to food items that are self-reported by a child in MEDAL but are not depicted in the reference photo, while an “omission” refers to food items that are depicted in reference photos but are not self-reported by the child in MEDAL. For instance, if a child self-reported consuming noodles in MEDAL, but this consumption was not observed in the reference photo provided by parents, this would be considered an intrusion. In contrast, if the reference photo showed that the child had consumed white rice, but the child did not self-report any food or drinks, this would be classified as an omission.

The formulas for calculating the match, intrusion, and omission rates are as follows [17]:

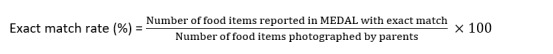

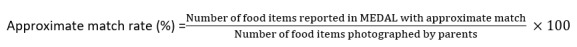

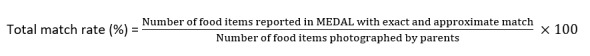

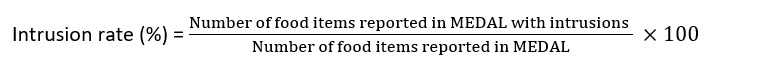

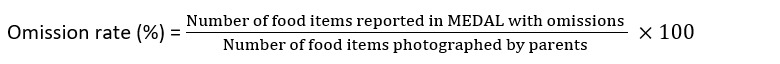











### Statistical Analysis

Descriptive statistics was used to summarize the characteristics of participants. The data were not normally distributed; therefore, the Mann-Whitney *U* test was used to test the differences in age between study 1 and study 2 participants. Pearson *χ*^2^ test and Fisher exact test were performed to compare the characteristics of study 1 and study 2 participants. The accuracy of self-reported food items was determined and expressed as median match rate (%), intrusions rate (%), and omissions rate (%). Furthermore, the Mann-Whitney *U* test was used to determine the difference in match, intrusion, and omission rates between the boys and girls. Kruskal-Wallis test was used to compare the median match, intrusion, and omission rates among different meals; then, the Bonferroni post hoc Mann-Whitney *U* test was used to identify the significant paired groups among the different meals. The match, omission, and intrusion rates by food group were presented as percentages (%). Statistical analysis was performed using the SPSS Statistics (version 25.0; IBM Corp) [22]. A significance level of *P*<.05 (95% CI) was adopted.

## Results

### Overview

Flowcharts of participants from both study periods are presented in Multimedia Appendix 1. A total of 53 students were recruited for both study periods, with 25 (47%) children in study 1 and 28 (53%) children in study 2. However, after excluding children who did not complete MEDAL or whose parents did not provide any food photographs, only 46 (87%) children were included in the analysis. All food photographs for all meals sent over the 4 days that corresponded to the MEDAL diet entries were included in the analysis. Only the food photographs or MEDAL diet entries of the eating occasions that were missing were excluded from the analysis.

Overall, 65% (30/46) of the participants were girls, 30% (14/46) were overweight and obese, and 82% (38/46) completed 3 to 4 days of recording in MEDAL (*P*=.76, *P*=.60, and *P*=.22, respectively; Table 1). However, a significant association was found among the ethnic groups between study 1 and study 2 (*P*=.004). A higher proportion of Malays (ethnic majority group) was found in study 1 than in study 2. This was because study 1 participants were recruited from national-type schools, while study 2 participants were recruited from national-type and Chinese schools. The median age of children in study 2 (median 10.8, IQR 10.56-11.26 years) was significantly higher than the median age of children in study 1 (median 10.2, IQR 9.9-10.4 years; *P*<.001). The higher median age of children in study 2 was due to study 2 being conducted near the end of the year. Differences in sex, weight status, and the number of days of MEDAL completion between participants in study 1 (21/46, 46%) and study 2 (25/46, 54%) were not significant (*P*=.76, *P*=.60, and *P*=.22, respectively).

**Table 1:** Characteristics of participants in study 1 and study 2 (n=46).

Characteristics		Total (n=46)	Study 1 (n=21)	Study 2 (n=25)	*P* value	
**Sex, n (%)**						.76^a^
	Boys	16 (35)	8 (38)	8 (32)		
	Girls	30 (65)	13 (62)	17 (68)		
Age (y), median (IQR)		10.53 (10.2-10.95)	10.2 (9.9-10.4)	10.8 (10.56-11.26)	<.001^b^	
**Ethnicity, n (%)**						.004^c^
	Chinese	18 (39)	3 (14)	15 (60)		
	Malay	25 (54)	16 (76)	9 (36)		
	Others	3 (7)	2 (10)	1 (4)		
**BMI status, n (%)**						.60^c^
	Underweight	3 (7)	2 (10)	1 (4)		
	Normal weight	29 (63)	14 (67)	15 (60)		
	Overweight	8 (17)	2 (10)	6 (24)		
	Obese	6 (13)	3 (14)	3 (12)		
**Number of days of completion of My E-Diary for Activities and Lifestyle, n (%)**						.22^c^
	<1	2 (4)	0 (0)	2 (8)		
	1	3 (7)	2 (10)	1 (4)		
	2	3 (7)	2 (10)	1 (4)		
	3	7 (15)	1 (5)	6 (24)		
	4	31 (67)	16 (76)	15 (60)		

^a^*P* values were assessed by the Pearson *χ*^2^ test (categorical).

^b^*P* values were assessed by the Mann-Whitney *U* test (nonparametric).

^c^*P* values were assessed by the Fisher exact test (categorical).

### Match, Omission, and Intrusion Rates

Table 2 presents the match (%), omission (%), and intrusion (%) rates observed between the food photographs received from the parents and self-reported food and drink items by the participants in MEDAL. The match, omission, and intrusion rates were 62%, 39%, and 20%, respectively. The total match rate was further broken down into exact match rate and approximate match rate, which were 60% and 1%, respectively. The median exact match rate (*P*=.03), total match rate (*P*=.004), and omission rate (*P*=.004) were significantly different among meal types. Of all meals, snacks showed a significantly lower total match rate (53%) than breakfast (83%) and dinner (75%; *P*=.01 and *P*=.02, respectively). Furthermore, snacks consumed were omitted more often (47%) than breakfast (18%) and dinner (25%; *P*=.01 and *P*=.02, respectively). Post hoc tests failed to indicate any significant difference among the meal types for exact match rate.

**Table 2:** Percentage of the match, omission, and intrusion rates observed between photographed meals and self-reported individual food and drink items in My E-Diary for Activities and Lifestyle (MEDAL).

Variables			Observed items^a^, n		Reported items^b^, n		Approximate match rate (%)^c^, median (IQR)		Exact match rate (%)^d^, median (IQR)		Total match rate (%)^e^, median (IQR)		Omission rate (%)^f^, median (IQR)		Intrusion rate (%)^g^, median (IQR)
On the basis of each meal (n=46)			1267		1004		1.4 (0-6.4)		60.3 (40.1-79.8)		61.6 (46-86.4)		39.3 (15.8-54.8)		19.5 (5.9-44.2)
**Meal types**															
	Breakfast (n=41)	330		301		0 (0-6.3)		76.3 (66.7-100)		82.5 (66.7-100)		17.5 (0-33.3)		0 (0-25)	
	Lunch (n=27)	329		215		0 (0-6.3)		64.3 (43.8-68.8)		64.3 (50-75)		35.7 (25-50)		0 (0-11.1)	
	Dinner (n=43)	526		445		0 (0-6.3)		75.0 (51.1-79.2)		75.0 (66.7-83.3)		25.0 (16.7-33.3)		4.2 (0-24.4)	
	Snack (n=21)	82		43		0 (0-0)		52.8 (25-100)		52.8 (25-100)		47.2 (0-75)		0 (0-50.0)	
	*P* value^h^	N/A^i^		N/A		.06		.03		.004		.004		.09	
**Sex^j^**															
	Boys (n=16)	440		339		4.0 (0-6.6)		49.2 (31-64.2)		53.0 (66.4-33.5)		49.1 (36.6-82.3)		29.7 (13-57.5)	
	Girls (n=30)	827		665		1.1 (0-6.8)		64.6 (48.6-87)		69.1 (55.9-87.2)		30.9 (13.3-44.1)		16.6 (4.4-32.5)	
	*P* value^k^	N/A		N/A		.94		.07		.03		.03		.26	

^a^Number of food items depicted in photographs.

^b^Number of food items reported in MEDAL.

^c^Approximate match is defined as a similar food with a slight variation, eg, full-fat milk and low-fat milk. Approximate match = (number of food items reported in MEDAL with approximate matches/number of food items photographed by parents) × 100.

^d^Exact match is defined as a selection of the same food or drink item in both the photograph and self-report in MEDAL. Exact match rate = (number of food items reported in MEDAL with exact matches/number of food items photographed by parents) × 100.

^e^Total match rate = (number of food items reported in MEDAL with exact and approximate matches/number of food items photographed by parents) × 100.

^f^Omission is defined as a food or drink item depicted in the photograph but not reported in the self-administered MEDAL report. Omission rate = (number of food items reported in MEDAL with omissions/number of food items photographed by parents) 100 = [omissions/(omissions + matches)] × 100.

^g^Intrusion is defined as a food or drink item reported in MEDAL but not depicted in the photograph. Intrusion rate = (number of food items reported in MEDAL with intrusions/number of food items reported in MEDAL × 100 = [intrusions/(intrusions + matches)] × 100.

^h^Kruskal-Wallis test (*P*=.05) and Bonferroni post hoc Mann-Whitney *U* test indicated that “snacks” had significantly lower total match than breakfast (*P*=.01) and dinner (*P*=.02) and higher omission than breakfast (*P*=.01) and dinner (*P*=.02).

^i^N/A: not applicable.

^j^Results show the rates for all meals for all 4 days.

^k^The Mann-Whitney *U* test (*P*=.05).

Compared to boys, girls reported more total matches (69% vs 53%) and fewer omissions (31% vs 49%; *P*=.03, respectively). There was no significant difference between boys and girls in exact match, approximate match, and intrusion rates (*P*=.07, *P*=.94, and *P*=.26, respectively; Table 2).

### Food Groups Reporting Accuracy

The match, omission, and intrusion rates of the food items were further analyzed by food groups (Table 3). At the food-group level, the “rice and porridge”; “breads, spreads, and cereals”; and “noodles, pasta, and potatoes” categories, the carbohydrate-based items, were reported most precisely (total match rates: 68%-76%). More than half of the “snack and dessert” items were omitted (omission rate: 54%). The side dishes that tended to be omitted by the participants were “vegetables and mushroom,” “eggs and tofu,” “meat and fish,” and “curry” (omission rates: 42%-46%). The food categories of “milk, cheese, and yogurt”; “snacks and desserts”; and “drinks” intruded most often (intrusion rates: 37%-46%).

**Table 3:** Percentage of matches, omissions, and intrusions observed between photographed meals and self-reported individual food and drink items in My E-Diary for Activities and Lifestyle (MEDAL) by food groups.

Food groups	Total observed items^a^, n (%)	Total reported items^b^, n (%)	Matches, n (%)				Omissions^c^, n (%)		Intrusions^d^, n (%)
			Total^e^	Approximate^f^	Exact^g^				
Rice and porridge (n=215)	195 (90.7)	168 (78.1)	148 (75.9)	10 (5.1)	138 (70.8)	48 (24.6)		20 (11.9)	
Fast food (n=36)	32 (89)	28 (78)	24 (75)	2 (6.3)	22 (68.8)	8 (25)		4 (14.3)	
Breads, spreads, and cereals (n=82)	73 (89)	61 (74)	53 (72.6)	2 (2.7)	51 (69.9)	21 (28.8)		8 (13.1)	
Noodles, pasta, and potatoes (n=82)	75 (91)	58 (71)	51 (68)	3 (4)	48 (64)	24 (32)		8 (13.8)	
Soups (n=38)	34 (89)	27 (71)	23 (67.6)	3 (8.8)	20 (58.8)	12 (35.3)		4 (14.8)	
Drinks (n=277)	201 (73)	209 (75)	131 (65.2)	10 (5)	121 (50.2)	68 (33.8)		78 (37.3)	
Milk, cheese, and yogurt (n=47)	31 (66)	35 (74)	19 (61.3)	0 (0)	19 (61.3)	12 (38.7)		16 (45.7)	
Fruits (n=71)	58 (82)	48 (68)	34 (58.6)	0 (0)	34 (58.6)	23 (39.7)		14 (29.2)	
Curry (n=24)	19 (79)	16 (67)	11 (57.9)	4 (21.1)	7 (36.8)	8 (42.1)		5 (31.3)	
Meat and fish (n=196)	187 (95.4)	116 (59.2)	108 (57.8)	13 (7)	95 (50.8)	79 (42.2)		9 (7.8)	
Eggs and tofu (n=95)	90 (95)	54 (57)	50 (55.6)	0 (0)	50 (55.6)	41 (45.6)		4 (7.4)	
Vegetables and mushrooms (n=191)	174 (91.1)	112 (58.6)	96 (55.2)	9 (5.2)	87 (50)	80 (46)		16 (14.3)	
Snacks and desserts (n=125)	98 (78.4)	72 (57.6)	45 (45.9)	0 (0)	45 (45.9)	53 (54.1)		27 (37.5)	

^a^Number of food items depicted in photographs.

^b^Number of food items reported in MEDAL.

^c^Omission rate = (number of food items reported in MEDAL with omissions/number of food items photographed by parents) [100 = omissions/(omissions + matches)] × 100.

^d^Intrusion rate = (number of food items reported in MEDAL with intrusions/number of food items reported in MEDAL) 100 = [intrusions/(intrusions + matches)] × 100.

^e^Total match rate = (number of food items reported in MEDAL with exact and approximate matches/number of food items photographed by parents) × 100.

^f^Approximate match rate = (number of food items reported in MEDAL with approximate matches/number of food items photographed by parents) × 100.

^g^Exact match rate = (number of food items reported in MEDAL with exact matches/number of food items photographed by parents) × 100.

## Discussion

### Principal Findings

Overall, the match, omission, and intrusion rates in this study were 62%, 39%, and 20%, respectively. We found that girls reported more total matches and made fewer omissions than boys. At the food-group level, carbohydrate-based items such as “rice and porridge”; “breads, spreads, and cereals”; and “noodles, pasta, and potatoes” were reported most accurately. However, “snack and dessert” items and side dishes of mixed meals such as “vegetables and mushroom,” “eggs and tofu,” “meat and fish,” and “curry” were omitted most often. Furthermore, items from “milk, cheese, and yogurt”; “snacks and desserts”; and “drinks” groups intruded most often.

This study indicated that Malaysian school children aged 10 to 11 years are capable of using the customized MEDAL web-based app to self-report food and drink items, albeit with some inaccuracies. While Tugault-Lafleur et al [18] stated that an acceptable cutoff of ≥85% for match rates and ≤15% for both omission and intrusion rates, this does not mean that the results from this study indicate poor accuracy for self-reported food items. The suggested cutoff was originally developed for meals in the school context. Furthermore, as Baxter and Thompson [23] suggested, children tend to recall food consumed as part of a 24-hour period with lower accuracy than a single meal. Children in this study reported all meals consumed in 24 hours at home and school rather than a single meal in the school context. Therefore, it is reasonable that the match rates would be lower and the omission and intrusion rates would be higher than the suggested acceptable cutoff values.

Furthermore, the total match rates (62%) from this study were slightly higher (better) than the total match rates from the validity study (60%) conducted in Singapore [21], although only school meals were included in the validity study conducted in Singapore and more meals were included in this study. However, food photography by parents was used as the reference measure, and thus there might be a possibility that the children in this study referred to the food photographs taken by their parents while self-reporting their meals in MEDAL. In addition, the match rates from this study were slightly higher than those from other studies that used web-based dietary assessment tools, for example, studies conducted in Brazil (43%) [16], Denmark (59%) [12], and the United States (42%) [24]. These studies used direct observation as the reference measure. Moreover, the slightly higher match rate in this study might be attributable to the fact that older children were recruited (aged 10 to 11 years) instead of the younger children (aged 7 to 9 years), as older children have better recall than younger children [25]. Furthermore, direct observation was used as the reference measure. In contrast, a Malaysian study [7] conducted among children aged 7 to 9 years demonstrated a higher match rate (89%) than this study, which might be attributable to the fact that the participants only needed to recall a single recess meal, whereas in this study, participants were asked to recall all their meals in a day.

The omission (39%) and intrusion rates (20%) in this study are higher than the cutoff value of ≤15% [18]. In general, children might have recorded their dietary intake in MEDAL the following day or later, instead of on the same day. According to Baxter et al [26], the accuracy of omission and intrusion rates declines significantly when the time interval between reporting and eating increases.

In addition, the omission rates (39%) were higher than the validity study conducted in Singapore (25%) [21] and other studies conducted in Malaysia (14%) [7], Brazil (28%) [16], Norway (27%) [17], and the United States (28%) [24]. These studies validated only lunches consumed in a school environment, which are fixed meals with little variation.

However, the increased omission rates in this study might be due to the inclusion of all meals consumed for validation purposes rather than a specific meal in a particular setting. This is further supported by “snacks” being the most omitted meal type in this study (47%). In conjunction with omission rates, “snacks and desserts” (54%) were food items that were omitted most often. This may also explain the higher omission rate in this study because children may be underreporting snacks and desserts, which are deemed to be unhealthy. Children may also forget to report snacks as one of their meals or they may be multitasking (eg, watching television or studying) while consuming snacks.

At the same time, carbohydrate-based items in “rice and porridge”; “breads, spreads, and cereals”; and “noodles, pasta, and potatoes” categories were reported most accurately (total match rates: 68%-76%) in this study. Carbohydrate-based items are usually consumed as main course items during each main meal in Asia, making them more recallable than side dishes or less common foods [5]. Moreover, this study found that the side dishes of mixed meals were commonly omitted, as items from the “vegetables and mushrooms,” “eggs and tofu,” “meat and fish,” and “curry” groups (omission rates: 42%-46%) were commonly consumed as side dishes, together with the carbohydrate-based items (eg, rice, porridge, or noodles) in Asia. Therefore, the results in this study are consistent with the findings of the study by Pérez-Rodrigo et al [5] that carbohydrate-based items are recalled better than side dishes. Children tend to recall salient items, such as main course foods, more easily than less salient items [27]. Side dishes of mixed meals were commonly omitted in this study because children may lack basic knowledge or familiarity with the food, food preparation, added ingredients, and components of side dishes in mixed meals [5]. Therefore, they omitted these food groups in their self-reports of dietary intake.

Furthermore, the intrusion rates in this study are slightly higher than those in the validity study conducted in Singapore (20% vs 15%) and a study conducted in Malaysia (3%) [7]. However, the intrusion rate is lower than those in studies conducted in Brazil (29%) [15,16] and the United States (30%) [24]. The food groups of “milk, cheese, and yogurt”; “snacks and desserts”; and “drinks” intruded most (intrusion rates: 37%-46%) in this study. These food groups intruded most because they are consumed along with the main meal or during snacking. These results are consistent with those of a study conducted in Brazil, where the intrusion rates are the highest for dairy products [16]. Furthermore, the results are supported by a study conducted in Norway, in which the items that intruded most were “yogurt” (56%) and “beverages and other” [17]. Despite this, the result of the intrusion is further influenced by the fact that parents might have forgotten to photograph the food items. Parents might have been unaware of their children consuming food or drink items along with their main meals or snacking and, thus, did not photograph the food.

Moreover, reporting accuracy was linked to gender differences. Girls often reported their dietary intake more accurately than boys [28]. The results in this study are consistent with the results from the study by Lyng et al [28], as the girls recalled better than the boys. The girls had a lower omission rate and a higher match rate than the boys (*P*=.03). This might be because boys are not as detailed as girls when selecting the food and drinks to report in MEDAL. Furthermore, a previous study demonstrated that compared to boys, girls recalled more details and provided more detailed elaborations on information content [29]. Therefore, the higher accuracy in reporting observed in girls may be attributed to their tendency to provide more detailed information. Furthermore, another study also showed that girls outperformed boys in object recall (recalling a series of pictures presented) and word selective reminding (a verbal free-recall task on word lists) than boys [30]. Consequently, it is plausible that girls exhibit better recall abilities regarding food and drink consumption, which could influence their selection of food and drink items in MEDAL.

Nevertheless, this study has several possible limitations. First, we used the parents’ photographs of the food items as the reference measure. The parents might have forgotten to photograph some food items, drinks, or snacks consumed by their children. Furthermore, parents might be unaware that their child had consumed certain food items, especially if they were working, which could lead to photographs of the food items not being taken. Thus, the intrusion rate was further affected by the forgetfulness or unawareness of parents. Another limitation was that the photographs of the food may serve as a visual-prompting tool for the children, which could increase the match rate. In addition, this study used a convenience sample, which was limited to schools in Kuala Lumpur city and students with internet access and electronic devices. Thus, the results of this study might not be extrapolated due to the limited technology skills in rural areas or other cities. Moreover, study 1 was conducted during the Ramadhan fasting period, which limited the meals reported by children during school recess. Therefore, overall, the results might not be generalizable to the non-Ramadhan fasting period.

Despite these limitations, this study has its strengths. First, it contributes to our understanding of dietary assessment among children aged 10 to 11 years in Malaysia. Furthermore, this is the first study conducted in Malaysia to determine school children’s ability to report dietary intake using a web-based diary. The main strength of this study is its validation of all meals consumed by the children in various settings instead of evaluating specific meals in a particular context. This enables the results to be extrapolated to meals consumed outside the school. As a result, numerous food and drink items were reported and analyzed, which increases the generalizability of the findings to the varied eating practices of a multicultural community.

This study has some implications for future research. In order to improve accuracy, children should be encouraged to report their food items in the web-based diary as frequently as possible and preferably on the same day. Furthermore, researchers should remind the children to report on the details of such food items as side dishes and snacks or drinks that are consumed along with meals or during multitasking. Future research should be conducted in different cities or rural areas to further affirm these results.

### Conclusions

In conclusion, the overall match (62%), omission (39%), and intrusion (20%) rates suggest that children are able to report their food and drink intake in MEDAL, although some items are omitted or intrude. Thus, MEDAL is a promising tool to assess the dietary intake of Malaysian school children aged 10 to 11 years. Future research should be conducted with a larger sample size and in nonurban areas to provide further insights into the accuracy of MEDAL in capturing the dietary intake of children.

## Appendix

Multimedia Appendix 1 Flowchart of participants in study 1 and study 2.

## Abbreviations

MEDAL: My E-Diary for Activities and Lifestyle

## Data Availability

The data sets used and analyzed during this study are available from the corresponding author on reasonable request.
