# Supplementary material for: Accuracy of a Web-Based Time-Use Diary (MEDAL) in Assessing Children’s Meal Intakes With Food Photography by Parents as Reference: Instrument Validation Study
Source: JMIR Pediatr Parent. 2024 May 7;7:e53461. doi: 10.2196/53461 (PMC11109862; doi:10.2196/53461)
Supplement: Multimedia Appendix 1 [file pediatrics_v7i1e53461_app1.docx]

**Multimedia Appendix**

**Eligible children (n = 173)**

**Excluded (n = 147)**

Did not return consent form (n = 142)

No internet at home (n = 2)

No device at home (n=3)

**Recruited (n = 26)**

**Excluded (n = 5)**

Child was absent (n = 1)

Children did not complete MEDAL at all (n = 2)

Parents did not provide any food photos (n = 2)

**Included in the analysis (n = 21)**

**Study 1 (April 2021)**

**Eligible children (n = 531)**

Received consent form in hardcopy (n = 142)

Received consent form through school’s newsletters or parents’ group (n = 389)

**Excluded (n = 473)**

Did not return hardcopy consent form (n = 116)

Did not fill up consent form in Google Form (n = 353)

Drop out before study (n = 4)

**Excluded (n = 33)**

Did not attend exit interview (n = 1)

Without food photography (n = 29)

Children did not complete MEDAL at all (n = 3)

**Recruited (n = 58 )**

Filled up consent form in hardcopy (n = 26)

Filled up consent form in Google Form (n = 36)

**Study 2 (October - December 2021)**

**Total participants included in analysis from both study period (n = 46)**

**Included in the analysis (n = 25)**

**Figure S1:** Flowchart of participants in Study 1 and Study 2
